# Patch tests and hand eczema: retrospective study in 173 patients and literature review^☆^

**DOI:** 10.1016/j.abd.2022.02.007

**Published:** 2023-03-02

**Authors:** Nathalie Mie Suzuki, Mariana de Figueiredo Silva Hafner, Rosana Lazzarini, Ida Alzira Gomes Duarte, John Verrinder Veasey

**Affiliations:** a Faculty of Medical Sciences, Santa Casa de São Paulo, São Paulo, SP, Brazil; b Dermatology Clinic, Santa Casa de São Paulo, São Paulo, SP, Brazil

**Keywords:** Allergic contact dermatitis, Hands, Patch tests

## Abstract

**Background:**

Hand eczema (HE) is a highly prevalent, recurrent, and multifactorial disease. It encompasses a group of eczematous diseases that affect the hands, etiologically classified into irritant contact dermatitis (ICD), allergic contact dermatitis (ACD) and atopic dermatitis (AD). Few epidemiological studies in Latin America have investigated the characteristics of patients with this condition and the origin of the disease.

**Objectives:**

To analyze the profile of patients diagnosed with HE submitted to patch tests aiming to determine its etiology.

**Methods:**

A retrospective descriptive study was carried out on epidemiological data and patch tests of patients with HE treated at a tertiary hospital in the city of São Paulo from January 2013 to December 2020.

**Results:**

A total of 173 patients were studied, whose final diagnosis was 61.8% of ICD, 23.1% of ACD and 5.2% of AD, with diagnostic overlap in 42.8% of the cases. The main positive and relevant patch tests were: Kathon CG (42%), nickel sulfate (33%), and thiuram mix (18%).

**Study limitations:**

: The number of treated cases and socioeconomic profile was limited to a vulnerable population group.

**Conclusion:**

HE is a diagnosis in which overlapping etiologies are frequent, with the main sensitizers identified in ACD being Kathon CG, nickel sulfate and thiuram mix.

## Introduction

The term ‘eczema’ comes from the Greek (*ekzein*) and means boiling. It is defined as an inflammatory disorder of the skin, clinically characterized by the presence of erythema, edema, infiltration, vesiculation, secretion, formation of crusts, scales, and lichenification. These lesions may follow one another or be associated, resulting in the polymorphous clinical picture of eczema.^1^ Histopathologically, eczema can lead to spongiosis, intracellular edema, acanthosis and parakeratosis in the epidermis and a dermal perivascular lymphocytic infiltrate. The main accompanying symptoms are pruritus, a sensation of burning and pain.

The term hand eczema (HE) encompasses a group of eczematous diseases that predominantly affect the hands and show a common clinical picture.

The classification of HE is still a controversial topic; it may be based on the morphology and etiology of the clinical lesions, but there is no universally accepted classification yet.

Regarding the morphology of the clinical lesions, HE can be classified as hyperkeratotic, erythematous-squamous, pompholyx (vesicular) and nummular. These presentations can vary dynamically throughout the clinical evolution of HE.

As for the etiology, it can be classified as irritant contact dermatitis (ICD), allergic contact dermatitis (ACD), atopic dermatitis (AD) and, less commonly, protein contact dermatitis. However, most of the time what is observed is an overlapping of these etiologies. Thus, HE commonly occurs in individuals with history of atopy and in those with history of contact with potentially sensitizing or irritating substances throughout their lives, often related to occupational exposure.

The estimated one-year prevalence of HE in Sweden is 9.7%.^2^ According to some studies, the incidence rate of HE is 5.5 new cases per 1,000 people per year, with a higher average incidence rate in females (9.6) when compared to males (4.0).^3^ This mainly occurs due to the higher frequency of AD in females and their greater exposure to sensitizing and irritating substances in occupational and domestic activities and not due to anatomical differences in the hands of the two sexes.

In addition to female gender, the main risk factors related to a higher incidence of HE are history of AD, which is the most relevant risk factor; the presence of a previous episode of HE, history of ACD, mainly related to sensitization to nickel sulfate; ‘wet work’, defined as one in which the hand is kept wet or when gloves are used for more than two continuous hours or when hand washing is carried out more than 20 times in one day; and age younger than 20 years at the onset.^4^ There is no clear association between HE and habits such as smoking and drinking alcohol or even the influence of the weather.^5^

HE is the most common occupational dermatosis. The main group affected by HE consists of women of working age, from 20 to 29 years of age. It affects 30% of women who occupy positions of high occupational risk for HE, the so-called “wet jobs”, such as healthcare professionals, cleaning and cooking professionals, hairdressers and workers in the manufacturing industry. The incidence of new cases of occupationally caused HE is estimated at 0.7 per 1,000 workers per year. However, the underreporting of cases can mask the real importance of this occupational problem, which can be 30 to 50 times greater.^6^ Studies show that HE is related to work absenteeism in 19.9% of cases and dismissal from employment in 23% of cases within a one-year period. In Sweden, a study showed that 82% of patients with HE had a change in work function due to the disease and 48% of them had to take time off from work at least once for a seven-day period due to the disease, and in 15% of cases, it culminated in unemployment or disability retirement.^7^ Finally, the cost of a patient with HE can range from €1,712 to €9,792 per year, mainly related to work absenteeism.^8^ To date, there are no similar studies in Brazil that quantified the cost of this disease.

In addition to the negative economic impact of the disease on the patient, there is also an important social stigma related to a visible dermatosis, which can cause a great burden. The hands are important for the expression and communication of individuals. Thus, any impairment of hand function or presentation can result in psychosocial damage, such as low self-esteem, social isolation and anxiety. HE is related to a negative impact on quality of life, similar to that found in patients with chronic diseases such as asthma and psoriasis.^9^ Moreover, these psychological factors can also negatively impact the disease.

The term chronic hand eczema (CHE) can be defined as the presence of HE for a period longer than 12 weeks or the recurrence of episodes at least twice a year.^4^ It can affect up to 9% of the adult population and has an estimated prevalence of 10% in one year in Scandinavian countries.^2^, ^3^ These are cases that are difficult to control and refractory to conventional treatments.

HE is a highly prevalent, commonly recurrent disease that affects the quality of life of individuals and also society economics. There are no recent studies characterizing the population profile in relation to the issue in Latin America. Thus, a study is necessary to characterize the population profile, which could contribute to the understanding of the issue in the studied country and provide relevant information so that, based on this information, effective prevention strategies can be outlined for the problem.

## Methods

This is a retrospective descriptive study carried out with the analysis of medical records and questionnaires applied systematically and routinely to patients with HE treated at the dermatology clinic of a tertiary hospital in São Paulo from January 2013 to December 2020. All adult patients of both sexes treated with clinical suspicion of HE who underwent patch testing were included in the study. Patients who had contraindications for undergoing the patch test (use of systemic corticosteroids for less than two weeks, sun exposure in the last 15 days, and presence of acute eczema); those who had incomplete or insufficient data in the medical records or in the outpatient questionnaire; those who did not attend the reading of the patch test in the 48 -h and 96 -h return consultations after its application; those who did not accept having their data included in the study; those who did not sign the informed consent form and pregnant women were excluded from the study.

The methodology of the patch tests applied to these patients is based on the application of allergenic substances that are fixed to the skin on the patient's back, with the help of chambers and adhesive tapes. The chambers used to fix the contact tests were AlergoChambers (Neoflex biotecnologia – São Paulo, Brazil).

All readings at 48 and 96 hours were performed by the same dermatologist who applied the tests, according to morphological criteria adopted by the International Contact Dermatitis Research Group (ICDRG) namely: (-) negative when no skin reaction is observed, and positive at three intensities, (+) slight erythema with some papules, (++) erythema, papules, and vesicles, (+++) intense erythema, papules and confluent vesicles.^10^

The test batteries used in this study were: the standard Brazilian battery (30 substances – FDA Allergenic, Rio de Janeiro, Brazil), cosmetic battery (ten substances – FDA Allergenic, Rio de Janeiro, Brazil), hair cosmetic battery (15 substances – IPI ASAC, São Paulo- Brazil), corticoid battery (seven substances - FDA Allergenic, Rio de Janeiro, Brazil), ulcerations battery (27 substances - Chemotechnique, Sweden). In some specific cases, some topical medications were produced in accordance with pre-established standards in the literature^15^ for the investigation of ACD to drugs.

The obtained data were entered into a Microsoft Excel spreadsheet for Mac, version 15.33, and the statistical analysis was performed using the SPSS v25.0 program.

## Results

During the period determined in this study, 184 people met the inclusion criteria, and 11 people met the exclusion criteria, resulting in a study population of 173 people.

Of the 173 assessed patients, 130 (75.1%) were female and 43 (24.9%) were male. The mean age of the studied group was 41 ± 15.46 years, with 43 ± 17.47 years for males, and 40 ± 14.65 years for females.

**Figure 1:**
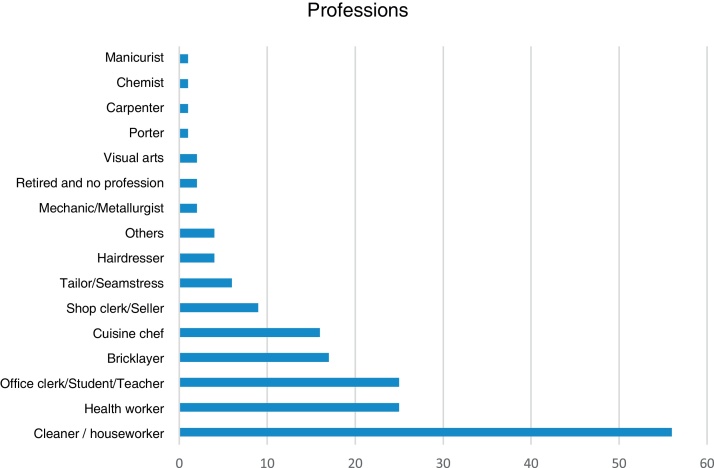
Distribution of professions in patients with HE.

As for occupations, 56 (32.4%) worked in cleaning/domestic services, 25 (14.5%) were health workers, 25 (14.5%) worked in offices or as teachers or students, 17 (9.8%) were bricklayers, 16 (9.2%) were cuisine chefs, nine (5.2%) were clerks or salespeople, 6 (3.5%) were tailors, four (2.3%) were hairdressers, four (2.3%) had other professions, two (1.2%) were mechanics or metallurgists, two (1.2%) worked with visual arts, two (1.2%) were retired or had no profession, one (0.6%) was a porter, one (0.6%) a carpenter, one was (0.6%) a chemist, one was a (0.6%) manicurist and one (0.6%) did not answer the question (Fig. 1).

As for housework, 146 (84.4%) reported doing some kind of housework, 20 (11.6%) denied doing housework and seven individuals did not answer the question. When asked whether they had contact with water for more than two hours a day, 82 (47.4%) said yes, 60 (34.7%) denied it and 31 (17.9%) did not answer the question. Regarding the act of washing their hands more than 20 times a day, 77 (44.5%) confirmed this habit, 65 (37.6%) denied it and 31 (17.9%) did not answer the question.

As for taking care of their clothes, 94 (54.3%) of the individuals mentioned washing clothes at home, 47 (27.2%) denied it and 32 (18.5%) did not answer the question. When asked whether they had a washing machine at home, 109 (63%) confirmed it, 32 (18.5%) denied it and 32 (18.5%) did not answer the question. As for washing dishes, 131 (75.7%) reported washing dishes at home, ten (5.8%) denied it and 32 (18.5%) did not answer the question. When asked whether they had a dishwasher at home, 16 (9.2%) confirmed it, 125 (72.3%) denied it and 32 (18.5%) did not answer the question.

Regarding the history of the disease, 79 (45.7%) reported a personal history of atopy, 71 (41%) a family history of atopy, and 79 (45.7%) a history of allergy to metals.

**Figure 2:**
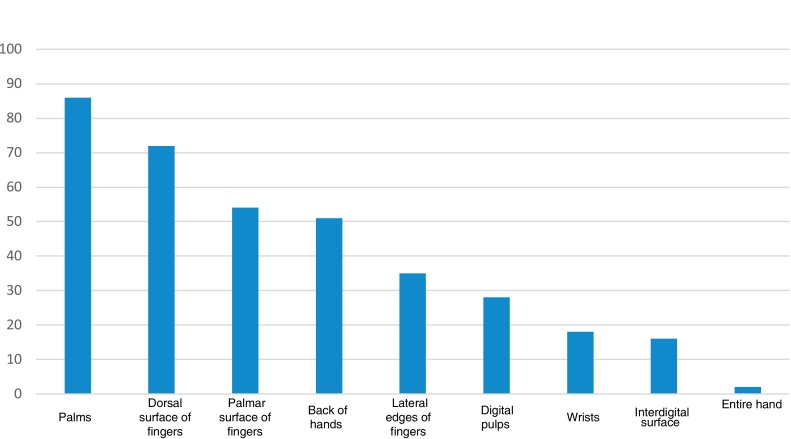
Hand distribution of HE.

As for the location of the HE, 86 (49.7%) had lesions on the palms, 72 (41.6%) on the dorsal surface of the fingers, 54 (31.2%) on the palmar surface of the fingers, 51 (29.5%) on the dorsum of the hands, 35 (20.2%) on the lateral edges of the fingers, 28 (16.2%) on the digital pulps, 18 (10.4%) on the wrists, 16 (9.2%) on the interdigital surface and two (1.2%) on the entire hand (Fig. 2).

As for the clinical presentation, 139 (80.3%) had desquamation, 137 (79.2%) erythema, 127 (73.4%) fissures, 43 (24.9%) vesicles and seven (4%) had pustules. Seventy-four (42.8%) of the individuals had lesions grouped into plaques.

Regarding lesions in areas other than the hands, 31 (17.9%) had lesions on the feet, 27 (15.6%) on the upper limbs, 14 (8.1%) on the legs, 13 (7.5%) on the face, seven (4%) on the scalp, seven (4%) on the cervical region, two (1.2%) on the chest, five (2.9%) on the back, three (1.7%) on the abdomen, three (1.7%) on the gluteal region and three (1.7%) on the thighs.

After the patch tests were performed, 98 (56.6%) cases were considered relevant and 75 (43.4%) were considered non-relevant for the current dermatosis, with previous or unknown relevance. The final diagnosis after the patch test was performed established that 107 (61.8%) of the individuals had a diagnosis of ICD, 98 (56.6%) had a diagnosis of ACD, 40 (23.1%) a diagnosis of AD, and nine (5.2%) individuals had other diagnoses. In this analysis, there was an overlapping of diagnoses in 74 (42.8%) patients. When analyzing these same 173 diagnoses isolated, that is, without cases with overlapping diagnoses, ICD was confirmed in 42 (24.3%) individuals, ACD in 41 (23.7%), AD in nine (5 .2%), and a different diagnosis in seven (4%) individuals (Table 1). Among all individuals, 98 (56.6%) of the cases were considered occupational dermatoses, whether irritative or allergic cases.

**Table 1:** Final diagnosis of HE.

Final diagnosis of HE	
A different diagnosis only	4%
AD only	5.20%
ICD only	23.70%
ACD only	24.30%
Two or more diagnoses	42.80%

Of the patients diagnosed with ACD, positive and relevant tests were observed for the following substances: Kathon CG in 41 (42%), nickel sulfate in 32 (33%), thiuram mix in 18 (18%), carba mix in 17 (17%), potassium bichromate in 17 (17%), cobalt chloride in 12 (12%), formaldehyde in 13 (13%), neomycin in ten (10%), methyldibromo glutaronitrile in six (6%), fragrance mix-1 in seven (7%), fragrance mix-2 in six (6%), palladium in five (5%), PPDA in three (3%), two cases (2%) for the substances balsam of Peru, lanolin, Mercapto mix, parabens, PPD mix, quartenium 15, quinoline, toluene resin and one case (1%) for the substances budesonide, bronopol, shellac wax, ketoconazole, colophony, decyl glucoside, diphenyl guanidine, dimethyl fumarate, Di(pentamethylene)thiuram disulfide, imidazolidinyl urea, triclosan, lauryl glucoside, promethazine, propylene glycol, tetraethylthiuram disulfide and tixocortol (Table 2).

**Table 2:** Positive and relevant tests in patients diagnosed with ACD.

Percentage of positive and relevant substances among individuals diagnosed with ACD		
Substance	n	%
Kathon CG	41	42%
Nickel sulfate	32	33%
Tiuram mix	18	18%
Potassium bichromate	17	17%
Carba mix	17	17%
Formaldehyde	13	13%
Cobalt chloride	12	12%
Neomycin	10	10%
Fragrance mix 1	7	7%
Methyldibromo glutaronitrile	6	6%
Fragrance mix 2	6	6%
Palladium	5	5%
PPDA	3	3%
Balsam of Peru	2	2%
Lanolin	2	2%
Mercapto mix	2	2%
Parabens	2	2%
PPD mix	2	2%
Quartenium 15	2	2%
Quinoline	2	2%
Toluene resin	2	2%
Budesonide	1	1%
Bronopol	1	1%
Shellac wax	1	1%
Ketoconazole	1	1%
Colophony	1	1%
Decyl Glucoside	1	1%
Diphenyl guanidine	1	1%
Dimethyl fumarate	1	1%
Dipentamethylenethiuram disulfide	1	1%
Imidazolidinyl urea	1	1%
Triclosan	1	1%
Lauryl glucoside	1	1%
Promethazine	1	1%
Propylene glycol	1	1%
Tetraethylthiuram disulfide	1	1%
Tixocortol	1	1%
**Total**	**42**	

## Discussion

In a general analysis of all patients with HE, it was observed that 75.1% of cases were identified in females, which coincides with the literature.^11^ It is suggested that the difference in prevalence between the sexes is essentially due to a difference in environmental exposure, both occupational and domestic, and not due to anatomical differences between the skin of men and women. The report of hand washing, for instance, is more frequent among women than among men. Such a habit has statistically significant association with the presence of HE according to the literature.^12^, ^13^

The present study identified a mean age of 40 years among women. However, the mean age of female patients with HE described in the scientific literature indicates a higher incidence in women aged 20 to 29 years, with a tendency to decrease with increasing age.^14^ A possibility for this divergence can be attributed to the environment in which the research was carried out: a tertiary public service in a developing country, where access to health care is unfortunately still limited, leading to delays in establishing the diagnosis and treatment of diseases.

As for occupation, the main professionals affected by HE in this study were: individuals working in cleaning or domestic services (32.4%), health workers (14.5%), and office workers, teachers, or students (14.5%). These findings coincide with those observed in the literature, in which the highest prevalence of HE over a period of one year occurs in: cleaning workers (21.3%), health workers (15.9%), and office workers (15.4%).^15^ Of all HE cases, 56.6% were considered occupational dermatoses, a higher percentage than found in the literature.^16^ These individuals were referred for evaluation by the occupational medicine team of the hospital since the work developed jointly by the specialties is of the utmost importance for the best management of these patients.

As for houseworkers, who do not fit into occupational dermatoses, but have several similarities with this situation, HE is more prevalent in the middle-income population when compared to the high-income one.^11^ The population in the present study comes from a public health service in a developing country, mostly with low family income. This characteristic is well demonstrated when one observes that only 9.2% of the patients reported having a dishwasher, 63% reported having a washing machine, and the majority of respondents (84.4%) carried out some type of domestic activity in their daily routines, such as washing dishes, cooking and washing clothes. The worsening of HE was also reported with contacts in 39.3% of individuals, including cleaning products, raw foods, water, and rubber gloves. It is clear that the need to carry out daily household activities, without having the option of delegating them to others, and the lack of household equipment that would reduce the time spent in contact with water during these activities probably contributed to the higher frequency of HE in this population, when compared with previous studies.^15^

In addition to everyday triggering factors, the patients background seemed to play an important role in the development of HE. The history of atopy is described in several studies carried out in general populations as the main risk factor and sign of severity for HE,^17^ which coincides with the findings described here, in which 45.7% reported a personal history of atopy and 41% reported a family history of atopy. AD is mainly related to HE in school-age individuals, where the frequency of atopy in individuals with HE can reach up to 90%.^18^ This relationship seems to become less relevant with age, but to date, there are no studies that determine the importance of this risk factor across different age groups.

Another interesting fact observed by some authors is that individuals with a history of atopy diagnosed with sensitivity to metals would be at greater risk for HE when compared to individuals who do not have sensitivity to metals.^12^

In the present study, 45.7% of the individuals reported some history of allergy to metals, a high frequency, and compatible with the literature. This relationship between metal sensitization and HE is still controversial, as it is currently unclear whether nickel sensitization would favor the onset of HE or whether the presence of HE would favor nickel sensitization.^19^

Moreover, the lack of a relationship between the etiology and clinical findings of HE has not been well established. The main clinical findings in the present study were desquamation and erythema, aspects that are common to all eczemas and coincide with the literature on the subject.^20^ It is believed that the clinical presentation of HE has a dynamic behavior, going through clinical presentations of acute, subacute and chronic eczema depending on the moment of its evolution, which is usually prolonged and very often recurrent. Thus, the clinical presentation would change over time in the same individual and would not be specifically related to a given etiology of HE.

As for the involvement of other areas of the body, a frequent relationship was found between the involvement of hands and feet, as 17.9% of HE cases also had similar lesions on the feet. According to the literature, this association may affect up to 30% of individuals.^16^ In these cases, the etiology is most often endogenous, and this finding has even been suggested as an etiological indicator.^21^ Some authors also believe that the higher frequency of dermatoses located on the feet such as tinea pedis associated with vesicular eczema of the hands could explain some of these cases.^22^

As for the etiology, ICD was the most frequent one, followed by ACD and AD, which is in agreement with the literature. The overlapping of diagnoses found in the present study in 42.8% of individuals is related to a worse prognosis and longer duration of the disease.^23^

Finally, it is worth discussing the results of the patch tests in this study; 43.4% of the positive tests were considered non-relevant, with prior or unknown relevance. Literature data report one-third of non-relevant positive tests, not accounting for prior or unknown relevance.^16^ The interpretation of the relevance of the patch test is of the utmost importance to correctly determine the etiology of HE. The application, reading, and interpretation of the tests must be carried out by a trained dermatologist so that there is no misdiagnosis. It is also important to remember that in addition to having a trained professional to carry out the test, the range of batteries available for the investigation is also very important.

The present study used ten complementary batteries, in addition to the standard Brazilian battery, and in specific cases, compounded substances. This initiative probably increased the rate of positive and relevant results compared to those in the literature^16^ which cannot be confirmed in this study since it was not its objective to compare this percentage of relevance, which requires further studies.

It is noteworthy that the choice of the complementary battery to be applied was individualized for each patient after a comprehensive anamnesis and physical examination during the first consultation. For instance, cases with clinical lesions on the face, scalp and cervical region may be related to ACD and components of shampoos, such as surfactants. Such components are only found in the hair cosmetic battery, one of the complementary batteries applied in these cases.

Regarding patients diagnosed with ACD, that is, positive and relevant patch tests, the main sensitizers found in the present study are in agreement with the literature, with nickel sulfate and preservatives occupying the top positions.^24^

Sensitization to metals is very common in the studied environment, with nickel being the main representative of this group. Previous studies carried out in the last 25 years at the same service as the present study indicate the same result, demonstrating that the high rates of metal sensitization have remained stable over these years.^25^, ^26^ One of the main sites for eczema in patients with nickel ACD is the hands, observed in 20.2% of sensitization in the present study and reaching up to 45% of cases in the literature.^27^ This contact can occur in different ways, occupational or not. One study showed that a frequent source of contact with nickel in Brazil are keys, which release both nickel and cobalt.^28^ Regulations regarding the release of nickel in products have existed in Europe since 1994, after the creation of the Nickel Directive, but these rules do not exist in Brazil, which maintains high exposure to this allergen for the Brazilian population.

Kathon CG, the commercial name given to the preservative consisting of the combination of methylchloroisothiazolinone and methylisothiazolinone in 3:1 parts,^29^ ranks second among sensitizers in the present study, being the preservative that caused most cases of ACD among HE patients. This preservative is widely found in personal care and cleaning products with liquid vehicles, such as shampoos, solutions, and gels. Historically, there has been an increase in sensitization rates, especially as of the year 2010, which led to restrictive measures first established in Europe to limit the concentration of this substance to up to 15 ppm for cosmetics that rinse off and to ban it from cosmetics that do not rinse off.^30^ This led to a reduction in the frequency of sensitization, but it still occupies one of the top positions among the sensitizers. In the times of the COVID-19 pandemic, when the use of soaps and gel alcohol increased, sensitization to these preservatives also became more evident, as it is found in many of these formulations. In August 2021, the National Health Surveillance Agency (ANVISA, *Agência Nacional de Vigilância Sanitária*) released a note stipulating the restriction of Kathon CG concentration at up to 15 ppm in personal hygiene products, cosmetics and rinse-off perfumes sold in Brazil, to be complied with before August 2024.^31^ This is expected to cause a reduction in the high sensitivity rates to this preservative still observed in Brazil.

Potassium bichromate, a chromium marker in patch tests, occupies the third place regarding frequency in the present study (17%). The world literature reports a high frequency of chromium sensitization in both HE and foot eczema cases attributed to the vast source of exposure to this substance, with leather being the main source.^32^ It is worth remembering that the legislation on the subject has been applicable in Europe since 2015, when the sales in the European market of leather articles that release more than 3 ppm of chromium and that come into direct contact with the skin were prohibited.^33^ Studies assessing the impact of these laws are not yet available, but a reduction in chromium sensitization is expected as a result of the legislation.

As for rubber vulcanizing agents, a group that also shows a high frequency of sensitization in the studied population, it was observed that the carba mix and thiuram mix groups showed a higher sensitization rate than the other groups, 18% and 17% respectively, compared to 2% in the PPD mix and Mercapto-mix groups. A high rate of release of substances from the thiuram group by rubber gloves is described in the literature, which would explain the greater sensitization in patients with HE when compared to patients with foot eczema, in which the main sensitizer is the Mercapto group, the ingredients of which are most commonly found in shoes and boots.^34^ Currently, there is a tendency to replace thiuram with dithiocarbamate in the manufacture of gloves for medical procedures, but yet, the thiuram-mix remains the main marker of ACD to dithiocarbamate, a fact justified by the similarity of their chemical structures.^35^ Still, regarding gloves, an increase in the use of diphenyl guanidine in the manufacturing of gloves for medical procedures has been observed in recent years, which would explain the greater sensitization found in the present study to the carba-mix group, to which diphenyl guanidine belongs.^36^

## Conclusion

The main clinical morphologies of eczema found in the present study were desquamation (80.3%), erythema (79.2%), fissures (73.4%), vesicles (24.9%), and pustules (4%). Lesions in plaques were found in 42.8% of the individuals. The predominantly affected site was the palms (49.7%). As for the factors related to the condition, 45.7% reported a history of atopy, and 45.7% reported a history of allergy to metals.

As for the final etiological diagnoses of HE, 61.8% of the individuals received a diagnosis of ICD, 56.6% received a diagnosis of ACD and 23.1% of the individuals received a diagnosis of AD. A total of 42.8% of the patients had two or more concomitant diagnoses.

In ACD cases, the main sensitizers were: Kathon CG 41 (42%), nickel sulfate 32 (33%), potassium bichromate 17 (17%), thiuram mix 18 (18%), Carba mix 17 (17%).

As for the cases of HE related to professional occupations, the most affected were cleaning professionals and those who perform domestic work (32.4%), health workers (14.5%), and office workers, teachers and students (14.5%).

## Financial support

None declared.

## Authors' contributions

Nathalie Mie Suzuki: Collection, analysis and interpretation of data; critical review of the literature, drafting and editing of the manuscript; critical review of the manuscript.

Mariana de Figueiredo Silva Hafner: Design and planning of the study, collection, analysis and interpretation of data; critical review of the manuscript.

Rosana Lazzarini: Collection, analysis and interpretation of data; critical review of the literature, approval of the final version of the manuscript; design and planning of the study; effective participation in research orientation.

Ida Alzira Gomes Duarte: Approval of the final version of the manuscript; design and planning of the study; effective participation in research orientation.

John Verrinder Veasey: Approval of the final version of the manuscript; design and planning of the study; effective participation in research orientation.

## Conflicts of interest

None declared.
